# Incentivizing Supplemental Nutrition Assistance Program Purchases with Fresh Produce in Corner Stores to Reduce Food Inequity in Underserved Areas of Washington DC

**DOI:** 10.1089/heq.2020.0028

**Published:** 2020-09-17

**Authors:** Anastasia M. Snelling, Jessica J. Yamamoto, Laura B. Belazis, Gabriella R. Seltzer, Robin L. McClave, Erin Watts

**Affiliations:** ^1^ American University Department of Health Studies, Washington, District of Columbia, USA.; ^2^ DC Central Kitchen Department of Food Access and Education, Washington, District of Columbia, USA.

**Keywords:** corner store intervention, fruit and vegetable consumption, minority health

## Abstract

**Purpose:** Maintaining a healthy eating pattern plays a key role in ensuring optimal health outcomes, yet, in areas considered “food deserts” and lower-income neighborhoods where the accessibility of healthy foods and beverages is limited, the pursuit of adequate nutrient intake is rendered cumbersome. This pilot program aims to improve access to healthful foods by supporting corner stores in stocking and promoting the purchase of produce.

**Methods:** DC Central Kitchen's Healthy Corners program in Washington, DC piloted a nutrition incentive model in 17 corner stores that were upgraded to stock an increased variety and quantity of fresh produce. This program, entitled “5-for-5,” provided a $5 coupon toward the purchase of fresh produce to Supplemental Nutrition Assistance Program (SNAP) shoppers making a qualifying purchase of $5 or more with SNAP benefits.

**Results:** Evaluation based on store owner buy-in and customer intercept surveys indicated overall satisfaction in program offerings with 77% of SNAP shoppers polled indicating an increase in produce consumption as a direct result of the program. Coupon distribution data indicated that in the 5-for-5 program's first year, 76.5% of all 57,989 distributed coupons were redeemed, amounting to $221,770 worth of incentivized fresh produce sales.

**Conclusion:** The results of the incentive program were promising with increases in the amount of produce purchased as a result of the program. Lessons learned concerning the use of a financial incentive to encourage the purchase of produce at corner stores is explored, as well as the feasibility of the corner store as a sustainable venue to increase produce consumption in underserved communities.

## Introduction

Health equity refers to an equal opportunity for all to pursue a higher quality of health, regardless of socioeconomic, demographic, or geographic background. To achieve health equity, individuals must be able to access wholesome foods that support a healthy lifestyle. The consumption of healthful foods, particularly fresh produce, is directly correlated with better health outcomes and lower risks for chronic diseases such as type 2 diabetes, heart disease, and obesity.^1^ With the rates of obesity still on the rise, models such as incentivized mobile and traditional farmers' markets and stocking corner stores with healthful foods are innovative strategies to overcome the decreased access to healthy foods in underserved communities.^2–5^

Low-income and racially diverse populations suffer from a higher burden of obesity and diet-related diseases and tend to have fewer full service supermarket options compared to other communities.^6,7^ Lower income neighborhoods have 25% fewer supermarkets when compared to middle-class neighborhoods, with the availability of supermarkets in predominantly African American neighborhoods being ∼50% less than white neighborhood counterparts.^8^ Furthermore, neighborhoods where fewer supermarkets are available tend to have a higher number of corner stores, which traditionally stock energy-dense, nutrient-poor foods.^9^

Health and nutrition researchers have advocated for increasing the consumption of healthy foods by making environmental changes to existing retail food outlets, including corner stores.^10–13^ Despite the limited availability of grocery stores, corner stores are often abundant in underserved communities. By using established food outlets, residents have the opportunity to incorporate increased access to healthy foods into their existing daily routine.^14^ Successful use of corner stores to effectively increase access to healthful foods will require advocates to consider more than just the issue of food access alone. It is imperative that interventions recognize and address the disparities that have contributed to the obesogenic climate in areas with reduced access to fresh foods, as outlined in the “Equity-Oriented Obesity Prevention Framework.” In accordance with this framework, interventions in vulnerable communities, where the availability of energy-dense foods outweigh the availability of nutrient-rich foods, would best serve residents by incorporating four key domains within their program models: (1) building upon community networks, (2) improving social and economic resources, (3) increasing healthy options, and (4) reducing deterrents or barriers.^15^ Although literature evaluating the impact of healthy corner stores remains stagnant, there is an affluence of documentation involving the process evaluation of healthy corner store initiatives. Cited barriers to the healthy corner store model include produce-stocking and meeting customer demands such as the need for reliability, cleanliness, and safety, as facilitators to corner store use are vast and include marketing, enhanced produce variety, and economical pricing.^16^

Existing interventions have placed focus on several key areas, including enhanced promotion of fresh foods through improved signage, food labeling, cooking demonstrations, and taste tests.^17^ One large scale “Healthy Corner Store Initiative” implemented by the Food Trust in Philadelphia, PA found the following to be a blueprint for success in increasing customer demand and store profitability when stocking produce at corner stores in underserved communities: (1) making improvements to store-infrastructure such as reliable refrigeration units and enhancing the inventory of fresh foods; (2) investments in Store Owners through technical training and incentives for participation, (3) engagement with the community through marketing, and (4) through corner store certification once the preceding steps were completed.^18^ An employment of similar strategies showed positive results in Columbus, OH with improvements seen in the amount of healthy items ordered, store traffic and purchases, and customer self-efficacy for healthful eating practices; and in Baltimore with increases in stocking and sales, among several other successful initiatives across the globe.^11,13,16,17^

In the United States, efforts have been placed on ensuring that firm linkages exist between food, food and nutrition policy, and agriculture.^19^ Yet, to meet fruit and vegetable intake goals set forth by Healthy People 2020, a report made in collaboration by the Health and Human Services Department, Center for Disease Control, and the Robert Wood Foundation suggests that pursuing continual connectivity between these three realms is a key component for federal policy and food assistance programs in their holistic effort to improve fruit and vegetable consumption across the United States. However, it is important that vulnerable urban populations also remain at the forefront of this effort, as the issue of obesity and disproportionate susceptibility to chronic illness strongly persists in affected communities, which are often lower-income areas, that are saturated with inexpensive energy-dense food options, with limited availability of economical fresh foods or compromised access to such items due to jeopardized neighborhood safety. Further strategies put in place to reduce food insecurity include the USDA Supplemental Nutrition Assistance Program (SNAP), which serves individuals and families that meet minimum income requirements for nutritional assistance. SNAP provides families the ability to increase their food budget by providing financial assistance for grocery shopping via a transfer of funds to an Electronic Benefit Transfer card, (EBT). SNAP benefits have been shown to reduce health care costs in lower income communities, yet, the program's impacts on fruit and vegetable consumption remain unclear.^20,21^ As an effort to increase produce consumption among SNAP participants, the Gus Schumacher Nutrition Incentive (GusNIP, formerly “FINI”) was forged to fund programs seeking to incentivize produce purchases made with SNAP funds across the United States while concurrently building and strengthening upon relationships between various stakeholders involved in food supply to create partnerships, which can better support lower income individuals and families. Grantees are challenged to create and test novel strategies to inform best practices in enhancing the purchase and incentivization of fresh fruits and vegetables with SNAP benefits.

As a grantee of the GusNIP grant, the current intervention offers an incentive to SNAP participants for the purchase of fresh produce at corner stores, with goals of improving the inequities found in the local food retail environment and tests a novel strategy of a 100% match of SNAP purchases in supported healthy corner stores with hopes of establishing ideal practices in the incentivization of produce. Corner stores have been noted as stable structures in the community and valuable contributors to the local economy, suitable for Healthy Corner Store conversion with modest subsequent improvements in the sales of healthy foods.^11,13,16,17^ Furthermore, outside of the Healthy Corner Store model, the incentivization of fresh produce purchases has also yielded significant results in sales data and inferred consumption of fruits and vegetables.^22,23^ Building upon these two successful models, in addition to the other aforementioned strategies, this intervention stands with very few pioneers in its successful incentivization of produce to SNAP participants in corner stores, taking a step toward improving local food access inequities, and to our knowledge is the first documented 100% match of SNAP dollars spent on fresh produce in corner stores while simultaneously empowering patrons with nutritional education. This intervention took place in some of Washington, DC's most vulnerable neighborhoods, where half of the District's SNAP recipients reside and access to full-service grocery stores is limited to approximately one store per 50,000 people.^24,25^ This pilot program, informed by the Equity-Oriented Obesity Prevention Framework, aims to empower store owners and enhance their ability to stock and promote fresh produce, and reduce barriers by meeting shoppers' needs for reliability and variety of produce while also encouraging healthful eating through building community resources and access.

## Methods

In 2011, the Healthy Corners program launched with goals of providing improved access to healthy foods in areas of Washington, DC with otherwise limited access, by provisioning highly frequented corner stores with fresh and vegetables. The Healthy Corners program, while simultaneously serving as a vendor for fresh produce, also included a number of different strategies (such as complimentary infrastructure upgrades and technical training) to entice store owners to support the sale of fresh produce while balancing the needs of the customer. As many corner stores are independently owned, the quantity of fresh produce necessary for purchase and distribution in an individual store does not often meet minimum standards for wholesale deliveries. The Healthy Corners program accordingly purchases mass quantities of fresh produce for participating stores in the Healthy Corners network, and then distributes the produce at wholesale prices under the stipulation that they are not to be marked up greater than 50–80% as outlined by DC Central Kitchen. In October of 2018, this program was expanded upon to establish a SNAP incentive program focusing exclusively on empowering urban corner store owners to stock and sell greater quantities of affordable, fresh produce to SNAP recipients while simultaneously improving the purchasing power of the shopper. The new program, entitled “5-for-5” included guidance for store owners on strategies to promote healthful food products through marketing and merchandizing techniques such as improved signage, infrastructure and food placement, as well as improved point-of-sales procedures and various community centered outputs such as cooking demonstrations, health and wellness fairs, and nutrition workshops.

This program model provided one $5.00 coupon for the purchase of fresh produce to shoppers who spent $5.00 in the store with SNAP benefits. Customers were eligible to receive one coupon per transaction, but there was no limit on the number of transactions or distribution frequency per customer. Store personnel were trained on the distribution of coupons on eligible transactions and the collection of transaction data. Each coupon had a unique number, expiration date, and a list of 4–7 nearby participating stores that were available for coupon redemption. Upon coupon distribution, clerks recorded the unique coupon number on the EBT receipt. At the time of redemption, the coupon was attached to the purchase receipt for later input into a spreadsheet for data analysis, followed by spot checks and sales comparisons to ensure data reliability. There was no limit to the number of coupons that could be redeemed, providing that they are redeemed before the expiration period of ∼4 months.

The 17 participating stores were selected specifically based on the following criteria: (1) ability to accept EBT, (2) store owner buy-in, as assessed by their engagement and dedication to the success of the Healthy Corners program, (3) shelf space available to accommodate an increased amount and variety of produce, (4) geographic location, (5) compliance with minimum stocking requirements, and (6) good standing with USDA Food and Nutrition Service. Once participating stores were selected, potential areas for infrastructure upgrades, improved product placement, and signage were identified and modified to accommodate increased produce visibility.

As a part of community outreach and program marketing, Healthy Corners program staff conducted a number of kick-off events and marketing campaigns to advertise the program. Monthly cooking demonstrations were held at several locations to empower shoppers on how to better use fresh produce. To further visually promote the program, banners were strategically placed at eye-level and marketing materials were posted on refrigeration units, windows, shelves, and check-out counters. Existing networks with community partners, nonprofit organizations, and low-income housing communities were also utilized to disperse flyers and program information.

As previous studies have indicated, essential to the success of a healthy corner store model is meeting needs of both the store owner and shopper. A survey of store owners to assess the program's success and learn about their experiences participating in the Healthy Corner program was collected at quarterly site visits by program officers. The survey included topics such as perceived customer demand for fresh foods, and whether they perceived the program to be good for business.

To ensure adequate reach to shoppers and community members, Healthy Corners also employed and trained three members of the community to serve as liaisons between Healthy Corner program staff, store owners, and the community. Each “Store Champion” resided within the neighborhoods that they served and covered three of the 17 stores, totaling 9 stores with a Store Champion on staff. Stores who were believed to welcome a Store Champion were selected to have a Store Champion on-site. Store Champions aimed to create a welcoming environment at corner stores by greeting customers, raising awareness for the program, and acting as a soundboard for program feedback. Feedback enabled swift modification for more tailored technical assistance in modifying how stores distribute and redeem coupons, and how to improve marketing materials to reach the intended audience. Further duties of Store Champions included attending hosted cooking demonstrations at local corner stores and community centers, delivering flyers to local partners, and collecting data regarding the behaviors and perceptions of shoppers through dialogue and customer intercept surveys. Institutional Review Board approval was obtained for this study in March 2019 (IRB:2019_308).

**FIG. 1.**
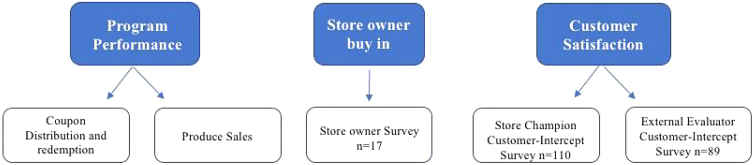
Program evaluation components.

The evaluation of this program was multipronged and included (1) sales and coupon data, including the number of coupons redeemed and the amount of produce sold to corner stores, (2) store owner buy-in and program satisfaction, and (3) customer intercept surveys: a collection of shoppers' perceptions and behaviors as evaluated by an external evaluator and the Store Champions (Fig. 1).

## Results

### Coupon distribution and redemption

**FIG. 2.**
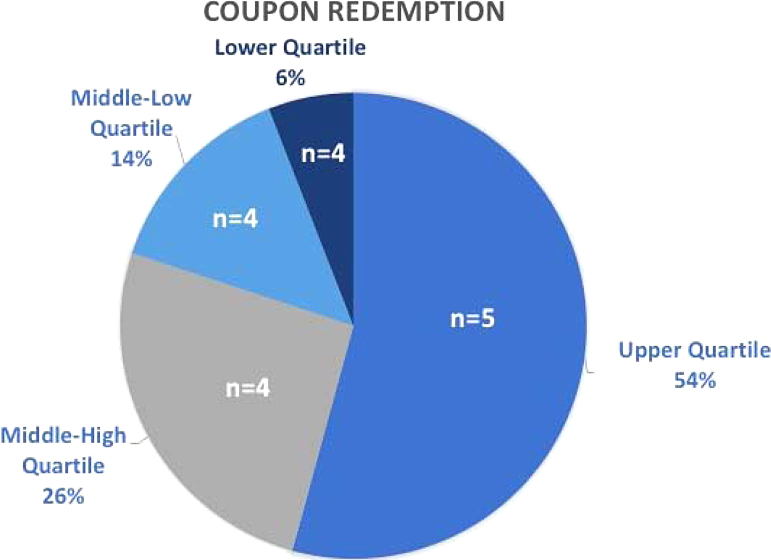
Coupon redemption by store quartiles.

Within 12 months, 44,354 of 57,989 distributed coupons were redeemed, a $221,770 and $289,945 value, respectively. The range of coupon redemption from highest-redeeming stores to lowest-redeeming stores was 6947 coupons with the top 2 quartiles of stores accounting for 80% of all 44,366 coupons redeemed (Fig. 2). This 76.5% redemption rate allowed for ∼8298 unique customers to be served. Seventy-seven percent of shoppers became repeat customers, utilizing the 5-for-5 program on multiple occasions. During this period (October 1, 2018 to September 20, 2019), 260,100 individual items of produce (including fruit cups as one item) were sold, which is a 288% increase from the 90,202 items sold in the previous unincentivized year.

### Store owner satisfaction and performance

Quarterly site visits captured the thoughts and perceptions of corner store owners. When posed the question, “How good for your business is selling fruits and vegetables?” 94.1% of store owners reported that the sale of produce was either “good” or “very good” when responding on a three-point Likert Scale ranging from “not good at all” to “good” to “very good.” Furthermore, 100% of store owner surveyed (*n*=17), indicated a customer demand for fresh produce. The store owner buy-in and attitude toward the Healthy Corners program was also assessed by program officers and ranked on a scale of 1–5, with 1 indicating minimal buy-in and 5 indicating a maximum level buy-in. Store owners participating in the 5-for-5 program, averaged a 4.53-point rating, indicating a perceived high level of commitment to their participation in the program.

### Customer intercept surveys—store champions

Store Champions conducted intercept surveys at the stores where they were based (*n*=9) to collect information regarding the purchasing preferences and behaviors of shoppers (*n*=110). Intercept surveys consisted of 10 questions and were administered verbally. Results indicated that while 70% of those surveyed purchase fresh foods at the given corner store, 92.3% of shoppers cited that the availability of fresh food at a store is a “very important” factor when deciding where to shop, alongside other factors, including personal safety (91.81%), cleanliness (94.54%), and accepting SNAP EBT (90.9%). Shoppers were able to select multiple items as “very important.” Three out of every four shoppers also indicated an interest in purchasing produce at the present corner store, and 66% of those polled indicated that lower prices and more frequent specials on fresh produce would lead to an increased likelihood of produce purchase.

### Customer intercept surveys—external evaluator

Customer intercept surveys were also conducted outside six stores to gather further insights into the impacts of the 5-for-5 program, two stores of which had a Store Champion on staff. Sixty-five percent of the shoppers surveyed (*n*=89) cited “almost daily” use of the corner store, and 79.8% of shoppers polled had purchased fresh produce at the present corner store. Among SNAP shoppers surveyed who were aware of the 5-for-5 program and have utilized the incentive, 77% of shoppers indicated an increase in their consumption of fruits and vegetables as a direct result of the 5-for-5 program, and 63% cited use of the incentivized purchase of produce on six or more occasions.

## Discussion

This study finds that the incentivization of fruit and vegetable purchases for SNAP-recipient corner store shoppers is a promising means to improve upon the access and affordability of fruits and vegetables in underserved neighborhoods. This is demonstrated by the near tripling of fruit and vegetable units sold from the previous year when the program was not available. Furthermore, the feasibility of the corner store as a critical component of the retail landscape, capable of improving the availability of fresh produce, was also further solidified in this study with findings that support the notion that many individuals visit corner stores on a near-daily basis, a point which may contribute to the high percentage of repeat customers utilizing the 5-for-5 program. Similar programs have been implemented at farmers' markets, yet, due to the lack of frequency and daily availability of farmers markets, repeat use of incentives has been shown to be a point of continued effort in some models.^26^

Consistent with current literature, it remains essential to empower both the store owners and shoppers alike. The store owner's role in the success and sustainability of the program remains critical in maintaining a positive environment and channel to distribute produce within the community.^18^ Further, the use of marketing and pricing appear to be facilitators for increased sale of fresh produce as well as improved signage and customer-oriented programs such as cooking demonstrations and food testing.^16,17^ Although process evaluations of the healthy corner store model have yielded positive results establishing best practices, continual evaluation into program impacts should remain a focus as many healthy corner store initiatives enter their tenure, to direct future initiative directions and funding to ensure an optimization of efforts, finances, and ultimately health effects.

The sustainability of this model is also founded in creating a firm demand for fresh produce that will remain postintervention, which would necessitate further training in the procurement of fresh produce. One viable option to enhance the postintervention sustainability is the creation of Corner Store Networks to cooperatively purchase produce, allowing for larger produce orders to meet wholesale requirements. Sustainability has also been enhanced in some models with financial support from funds collected from imposed soda taxes, which may be a plausible future direction for the present model.^27^

Corner stores remain a stable part of the local economy, often passed down from generation to generation and fall under the category of “convenience stores,” which accounted for 34% of the brick and mortar retail offerings in the United States, and 3.1% of gross domestic product in 2017 (yet, convenient stores which sell fuel account for 80% of stores in this grouping).^28^ While it has been found that many people visit corner stores on a near daily basis, reduced access to such venues has been shown to reduce the obesity risk. However, it is possible to change this narrative by improving the offerings of healthy products at corner stores, which has shown potential for increasing the purchase of healthy foods by allowing shoppers to incorporate healthy foods into their daily routine, especially when emphasis is placed on product pricing, nutritional education, and promotional strategies.^16,18^ In determining promising inputs for future incentives and investments, we found it essential to examine the top-performing stores as identified by their distribution and redemption of coupons, as compared to their lower distributing and redeeming counterparts. A clear trend was revealed upon examination of the top 2 quartiles of higher performing stores. Stores with a “Store Champion” and a higher investment to infrastructure and space dedicated to produce were observed to be two traits of most high-performing stores. The presence of Store Champions appears to support the program success, although this factor is also intertwined with the presence of store owners who are also committed to the success of the program. However, we hypothesize that engagement of the community on an individual level, by neighbors employed as Store Champions may have aided in building trust in the program, in addition to allowing more personalized marketing. Currently, the purchase of fresh foods at corner stores may be seen as a risk since such products have not been traditionally offered at these venues. Shifting the views of the shoppers to the corner store being a place for reliable fresh food may take time, as well as investment. Reinforcing positive perceptions by the consumer through a low-risk incentive, endorsed by members of their community may be a potential bridge between the lack of trust in corner store produce and may slowly begin to instill a belief in the quality and reliability of fresh offerings being able to be a part of the corner store panorama, while simultaneously giving rise to demand for fresh foods and the purchasing power of the consumer.

Programs such as the present study, which offer a 100% subsidy toward the purchase of fresh produce, also offer improved access points to fresh foods within one's daily routine, which is especially important in a situation of national pandemic where resources and means of transit are limited. A means for significant improvements in health equity in underserved communities are also presented in this model. A recent study utilizing a simulation model found that the incentivization of SNAP products with a subsidy of 30% for fruits and vegetables could lead to a reduction of type 2 diabetes, myocardial infarction, stroke, and obesity by 1.7%, 1.4%, 1.2%, and 0.2%, respectively, and save ∼12,000 lives from cardiac failure.^29^

The solutions to the problem of inequity in underserved communities are just as vast as the determinants, which have led to the present disproportionate services to the public; however, this study serves as one piece of the puzzle in bringing equity to the food retail environment and potential clues for the optimization of fruit and vegetable incentivization.

## Limitations

Limitations to this study include an inferred consumption of fruits and vegetables based on produce purchase and customer recollection. Current data available only allow for the observation of produce purchase and not waste. In addition, the present study was conducted in Washington, D.C. in a subset of corner stores and may not be able to be extrapolated to other geographic locations or SNAP participants.

## Conclusions

Corner stores are often regarded as a community staple. Given their abundance in underserved communities, many public health advocates seek to utilize these existing venues as a channel for improving access to fresh foods. Modifying perceptions of the corner store may take time as does individual behavior change; yet, providing fruits and vegetables with minimal financial risk to shoppers can be an opportunity to shift perceptions of the quality and availability of corner store offerings. Corner stores are only one strategy to bring fresh produce and healthier foods into underserved neighborhoods, but they appear to be a viable outlet that will need continued support to sustain this effort.

## Abbreviations Used

EBT: Electronic Benefit Transfer card
SNAP: Supplemental Nutrition Assistance Program
